# Elucidating the Function of Penetratin and a Static Magnetic Field in Cellular Uptake of Magnetic Nanoparticles

**DOI:** 10.3390/ph6020204

**Published:** 2013-02-06

**Authors:** Suman Chaudhary, Carol Anne Smith, Pablo del Pino, Jesus M. de la Fuente, Margaret Mullin, Andrew Hursthouse, David Stirling, Catherine C. Berry

**Affiliations:** 1 Centre for Cell Engineering, Joseph Black Building, University of Glasgow, Glasgow, G12 8QQ, UK; E-Mail: c.a.smith@bio.gla.ac.uk; 2 Integrated Microscopy Facility, Joseph Black Building, University of Glasgow, Glasgow, G12 8QQ, UK; E-Mail: M.mullin@glasgow.ac.uk; 3 Instituto de Nanociencia de Aragon, University of Zaragoza, Edif I+D, C/ Mariano Esquillor s/n, 50018 Zaragoza, Spain; 4 School of Science, University of the West of Scotland, Paisley, PA1 2BE, UK; E-Mail: andrew.hursthouse@uws.ac.uk (H.A.); david.stirling@uws.ac.uk (D.S.)

**Keywords:** penetratin, magnetic field, nanoparticles, clathrin, endocytosis, ICP-MS, TEM

## Abstract

Nanotechnology plays an increasingly important role in the biomedical arena. In particular, magnetic nanoparticles (mNPs) have become important tools in molecular diagnostics, *in vivo* imaging and improved treatment of disease, with the ultimate aim of producing a more theranostic approach. Due to their small sizes, the nanoparticles can cross most of the biological barriers such as the blood vessels and the blood brain barrier, thus providing ubiquitous access to most tissues. In all biomedical applications maximum nanoparticle uptake into cells is required. Two promising methods employed to this end include functionalization of mNPs with cell-penetrating peptides to promote efficient translocation of cargo into the cell and the use of external magnetic fields for enhanced delivery. This study aimed to compare the effect of both penetratin and a static magnetic field with regards to the cellular uptake of 200 nm magnetic NPs and determine the route of uptake by both methods. Results demonstrated that both techniques increased particle uptake, with penetratin proving more cell specific. Clathrin- medicated endocytosis appeared to be responsible for uptake as shown *via* PCR and western blot, with Pitstop 2 (known to selectively block clathrin formation) blocking particle uptake. Interestingly, it was further shown that a magnetic field was able to reverse or overcome the blocking, suggesting an alternative route of uptake.

## 1. Introduction

Nanoparticles (NPs) are sub-micrometer sized particles, typically less than 500 nm [1]. Due to their small size they have the capability to cross the highly electrically resistant blood brain barrier as well as tight junctions [2]. Recent advances in nanotechnology allow functionalisation of NPs with ligands for various therapeutic and diagnostic applications including bio-sensing, magnetic resonance imaging (MRI), site-specific drug delivery, stem cell tracking and treatment of hyperthermia [3]. Several NP attributes contributing to these applications include: high surface area to volume ratio and high surface reactivity [4]. Magnetic NPs (mNPs), such as superparamagnetic iron oxide nanoparticles (Fe_2_O_3_ and Fe_3_O_4_), are of such a size that they are easily magnetised under an applied field, but lose their magnetism as soon as the magnetic field is removed (thus preventing NP aggregation), and provide an excellent platform for use in clinic. Currently mNPs are employed in clinic in MRI, which allows intra-tissue and intracellular detection. 

The major hurdle presented to cellular internalisation is the cell plasma membrane, which acts as a barrier to block the molecules that are usually not actively imported by cells. This selective permeability of the cell membrane is due to the presence of the lipid bilayer, which is interspersed with transmembrane proteins. The hydrophobic nature of these lipids prevents the diffusion of various polar solutes such as proteins and peptides across the cell membrane, leading to the prevention of unconstrained influx and efflux of various solutes [5]. 

When contemplating NP delivery into cells, the main cell uptake routes are: (i) specific uptake, such as receptor-mediated endocytosis, including both clathrin- and caveolin-mediated and (ii) non-specific uptake, typically pinocytosis (or ‘cell drinking’) [6]. Caveolae are typically 50-100 nm sized flask- shaped membrane pits, formed by the inward budding of plasma membrane, that are found on the surfaces of smooth muscle cells, endothelial cells and fibroblasts, and are involved in clathrin-independent endocytosis [7]. Alternatively, clathrin-dependent endocytosis is a receptor-mediated endocytosis, which involves the formation of plasma membrane vesicles *via* membrane invagination, containing receptors that are highly specific to the molecule being internalised. It involves the recruitment of the endocytic structures that are stabilized by clathrin triskelia that form the clathrin coat, and contain a N-terminal β-propeller domain (TD) that acts as a hub for protein-protein interaction [8].

These routes have varying degree of success as any drug or DNA cargo attached to the NP are ultimately localised in cytoplasmic endosomes, and are thus open to cell degradation [6]. To overcome this difficulty, and to further enhance the cellular uptake, NPs can be conjugated with cell penetrating peptides (CPPs), which are vectors employed for to enhance cell internalisation [9,10]. CPPs are short peptides containing a protein transduction domain, usually confined to less than 20 amino acids, conferring the ability to cross the cell membrane [11]. The common structural feature of these CPPs is the presence of basic or cationic amino acids, in particular lysine and arginine, conferring the translocation properties [12]. The first discovered CPP was the HIV transactivator of transcription (tat) peptide, which is a non-toxic curtailed version of the naturally existing protein [13,14]. Dowdy *et al.* suggested in 2001 that they are basically charged [15], with 48-60 amino acid residues being mainly responsible for the translocation properties of the membrane [16]. Other, naturally occurring CPPs include the 60 amino acid homeodomain of *Drosophilla* Antennapedia [17,18] and herpes simplex virus type 1 protein [19], both of which are able to translocate the cell membrane. The discovery of these natural peptides was followed by the development of various synthetic analogues of tat peptides such as penetratin, a peptide of 16 amino acids, derived from the DNA binding domain of the Antennapedia homeoprotein, which is currently the second most commonly used CPP in NP research after the HIV tat peptide [20].

In addition to the use of CPPs, the efficacy of mNP internalisation may be further enhanced by the use of external static magnetic fields [21] through a process termed ‘magnetofection’, which is capable of increasing the transfection rates to about 100 fold using extremely low concentrations of mNPs [22].We have previously shown that both the use of magnetic field and penetratin increased cellular uptake of 500 nm NPs [23]. In addition, a recent study in our group demonstrated that when considering cells cultured in 3D (collagen gel culture), a magnetic field was complimentary to the use of 200 nm mNP functionalised with CPPs, with both together providing the optimal gel penetration and cellular uptake as opposed to when used alone [24]. However, the method of cell uptake in response to both penetratin and a static field and whether the cell indeed uses a different uptake mechanism for either, are unknown.

Therefore, this paper employs two different blockers of endocytosis, Pitstop 2 and Dyngo 4a, in a bid to determine the cellular uptake route. Pitstop 2 impedes the receptor-mediated endocytosis by arresting the clathrin-coated pit dynamics at multiple stages [10], whilst Dyngo 4a is a structural analog of Dynasore with increased efficacy and is a potent therapeutic target, for the treatment of Botulism as well as various other diseases involving the dynamin-dependent uptake mechanism [25]. In the study herein, fluorescently tagged 200nm mNPs functionalised with a penetratin were introduced to monolayer fibroblast cells in the presence and absence of a magnetic field. Cell internalisation was quantified, and the mechanism of uptake was analysed using PCR and western blot (clathrin and caveolin) alongside internalisation when cultured with the specified blockers. Results demonstrated that while both penetratin and the field significantly increased internalisation, with the former proving more cell-specific, both also appeared to employ clathrin-mediated endocytosis as a means of uptake. It was noted that the magnetic field could overcome the specific blocking action, suggesting an alternative route of uptake.

## 2. Experimental Section

### 2.1. Nanoparticle Synthesis and Functionalisation

For the derivatisation of 200 nm amine terminated magnetic green-fluorescence nanoparticles (nano-screenMAG/G-PEA) with penetratin, we adapted previously reported protocols [24,25]. Given that the derivatization approach requires coupling of amine terminated materials and sulfhydryl containing peptides (penetratin), sulfosuccinimidyl-4-(*N*-maleimidomethyl)cyclohexane-1-carboxylate (sulfoSMCC) was used as crosslinker. Sulfo-SMCC is a heterobifunctional crosslinker containing an amine-reactive N-hydroxysuccinimide ester on one end and a thiol-reactive maleimide group on the other end. Amine-containing NPs may be activated with sulfo-SMCC to derive sulfhydryl-reactive maleimides, aiming conjugation with thiol-containing molecules as in the here reported case, i.e. activated penetratin. The following protocol describes in detail the derivatization approach used herein:

First, amine-terminated NPs (290 µL from a 35 mg/mL stock solution) were added to freshly prepared ice-cold *buffer-A* [100 mM 2-(N-morpholino)ethanesulfonic acid (MES), 150 mM NaCl, pH 7.2] containing the sulfoSMCC crosslinker (500 µg). The sulfoSMCC activation reaction was allowed to proceed for 30 min at room temperature. Then, activated NPs were magnetically precipitated, that is, excess crosslinker was washed out. Please, note that these magnetic NPs can be magnetically precipitated using a standard magnetic separator (MagnetoPURE, Chemicell). Activated NPs were suspended in ice-cold *buffer-A* containing activated penetratin, enabling coupling overnight at 4 °C. Uncoupled peptide was washed out by two steps of magnetic precipitation as previously described. Finally, peptide-derivatized NPs were dissolved in 100 mM MES, adjusting the final volume to 1 mL (see Table 1).

**Table 1:** Chemical description of the 200 nm NPs after functionalisation with penetratin. The concentration of NPs in a 1 mL sample is displayed in both mg and nmoles. The molar ratio of mNPs to sulfo-SMCC, (which was used for amine activation) is shown. The concentration of penetratin in each 1 mL sample and the molar ratio of mNPs to penetratin is shown.

	mNPs (mg)	mNPs (nmoles)	~ Molar ratio (mNP:sulfoSMCC)	Penetratin (nmoles)	~ Molar ratio (mNP:Penetratin)
nano-screenMAG/G-PEA – 200 nm	10	3.7 × 10^-3^	[1:310 10^3^]	29	[1:7.8 10^3^]

### 2.2. Transmission Electron Microscopy NP Characterisation

A 2 μL drop of NP solution at a concentration of 0.1 mg/mL in milliQ H_2_O was dried onto a carbon coated grid and viewed under a Leo 912 AB TEM at 120kV, at 40,000× magnification.

### 2.3. Cell Culture

Infinity Telomerase Immortalised primary human fibroblasts (hTERT-BJ1, Clontech Laboratories, Inc., California, USA) were seeded on to glass coverslips (13 mm diameter) at a density of 1 × 10^4^ cells per disk in 1 mL of complete medium. The medium used was 71% Dulbecco’s Modified Eagles Medium (DMEM; Sigma, Dorset, UK), 17.5% Medium 199 (Sigma), 10% Fetal Bovine Serum (FBS; Lonza, Castleford, UK), 0.9% 100 mM sodium pyruvate (Life Technologies, Paisley, UK). The cells were incubated at 37 °C with a 5% CO_2_ atmosphere for 24 hours (for fluorescence, ICP-MS), or 5 days (for q-rtPCR, Western Blotting, TEM) to ensure cell confluence. At this point cells were incubated with NPs for 30 minutes (0.1 mg.mL^-1^), unless specified otherwise, at 37 °C, 5% CO_2_ +/- 350 mT magnetic field (magnetoPLATE, Chemicell GmbH, Berlin, Germany). Control cells were cultured in the absence of NPs, +/- 350mT magnetic field. With regards to endocytosis blocking experiments, cells were incubated with both Pitstop 2^Tm^ and Dyngo 4a^Tm^ blockers (Abcam, Ascent Scientific, Cambridge, UK) at the recommended concentration of 30µM for 15 min (37°C, 5%CO_2_) prior to incubation with the NPs (+/- 350 mT magnetic field). Control cells were cultured in the absence of both Pitstop and Dyngo blockers.

### 2.4. Quantification of NP Uptake into Cells: Inductively Coupled Plasma Mass Spectrometry (ICP-MS)

Cells were grown in a 96 well plate and then incubated with the NPs. Incubation was performed +/- a magnetic field, for either 1 or 18 hours. After incubation, the media from each sample was removed and added to 1 mL of H_2_O, together with 100 μL PBS that had been use to wash the cells and wells. Samples were treated with 1 mL of 70% nitric acid (analytical reagent grade, S.G 1.42, Fisher Scientific, Loughborough, UK) and then heated in a water bath at 70°C overnight. After heating, samples were cooled and then diluted to 50 mL with H_2_O (MilliQ H_2_O used throughout). Samples were analysed using inductively coupled plasma mass spectrometry (ICPMS; THERMO X Series II, Thermo Fisher Scientific, Loughborough, UK), by monitoring mass 56 in hexapole CCT ED (Collision Cell Technology with Energy Discrimination) mode to minimise interference from argon oxide. Quantification of iron using calibration solutions prepared from Spex "CertiPrep" standards diluted as required with 1% v/v trace element grade nitric acid (Fisher Scientific, Trace Metal Grade). Calibration included 6 standards in the range 0-1000 ng/mL with In-115 internal standard at 100 ng/mL. Three replicates were collected per sample. Statistical analysis was performed in SPSS. The ICP-MS data for each size of mNP (100 or 200 nm), for each time point (1 or 18 hours) was analysed by 1-way ANOVA with Dunnett’s test [n = 3].

### 2.5. Cell Internalisation of NPs

Cell were incubated with the NPs for 5 minutes, 30 minutes and 60 minutes at 37 °C, 5% CO_2_ +/- 350 mT magnetic fields to determine uptake patterns with time in culture. Control cells were cultured in the absence of any NPs, +/- 350 mT magnetic field. Cells were washed with PBS, fixed with PBS/4% formaldehyde and washed again with PBS.

#### 2.5.1. Fluorescence Microscopy

Coverslips were mounted with 4’6-diamidino-2-phenylindole (DAPI, in fluorescent mounting medium, Vector Laboratories Inc. Burlingame, UK) before being viewed using a Leica DM IRB fluorescent microscope. 

#### 2.5.2. Fluorescent Platereader

The samples were washed several times in PBS prior to fluorescent detection on the Modulus^TM^ Microplate Multimode Reader (Turner Biosystems, Promega, Southampton, UK) using the blue filter (excitation 490 nm, emission 510-570 nm), which had spectra closest to that of the NPs.

### 2.6. Route of NP Internalisation

#### 2.6.1. Clathrin and Caveolin Real Time Polymerase Chain Reaction (q-rtPCR)

Cells were as described on a 24 well plate and challenged with the NPs. After 1 hour, treatments were removed and RNA was extracted using an RNeasy Mini Kit (Qiagen). Reverse transcription was performed using a QuantiTect Rev. Transcription kit (Qiagen). Each qRT-PCR reaction contained 10 ng cDNA. Three technical replicas were used for each of three biological replicas. A 7500 Realtime PCR System (Applied Biosystem, Life Technologies, Paisley, UK) was used, with Taqman master mix and Abi Prism 96-well plates as supplied by Applied Biosystems. Primers and probes for clathrin and caveolin and GAPDH were supplied by Eurofins MWG Operon (Germany, Europe)

#### 2.6.2. Western Blotting Analysis of Clathrin and Caveolin

Following NP incubations 1 mL PBS was added per sample, samples were lysed using three freeze/thaw cycles of -80 °C for 20 minutes followed by 37 °C for 30 minutes. Total protein concentration of each sample was determined spectrophotometrically using the Nanodrop (Labtech, East Sussex, UK). Equal concentrations of all samples were reduced, then run on a 4-12% NuPAGE Novex Bis Tris Gel using the NuPage electrophoresis system, following manufacturer’s instructions. Proteins were then transferred to Hybond-P PVDF Membrane following manufacturer’s instructions. After non-specific blocking, detection of clathrin and caveolin was performed using specific antibodies and the ECL Western Blot System. X-ray film was exposed for 1 minute prior to developing using Kodak developer and fixer solutions.

#### 2.6.3. Transmission Electron Microscopy (TEM)

Following magnetic NP incubation, samples were fixed using 1.5% glutaraldehyde (Sigma) buffered in 0.1 M sodium cacodylate (Agar Scientific, Stanstead, UK) at 4 °C for 1 hr and postfixed with 1% osmium tetroxide (Agar) and stained with 0.5% uranyl acetate for 60 min. Samples were then dehydrated through a series of ethanol concentrations (30%, 50%, 70%, 90%) up to dried absolute alcohol, followed by three changes in propylene oxide. Subsequently the coverslips were treated with 1:1 propylene oxide:resin (TAAB Araldite epon resin) and incubated overnight in order to evaporate the propylene oxide. The next day, coverslips were embedded in pure resin and cut in ultrathin sections. Finally, the samples were stained with 2% methanolic uranyl acetate for five minutes and Reynolds lead citrate for five minutes, followed viewing under a LEOG12AB transmission electron microscope at 120 kV.

## 3. Results and Discussion

### 3.1. NP Characterisation

**Figure 1:**
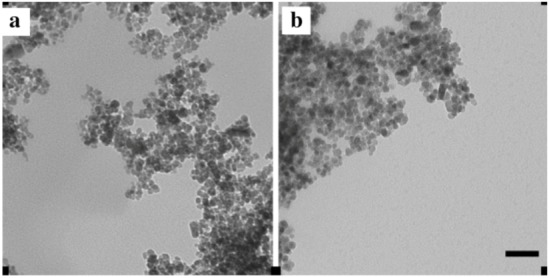
TEM images of the 200 nm plain (a) and 200 nm penetratin-functionalised (b) NPs. [Scale bar = 100 nm].

The TEM images of the NPs are shown in Figure 1. The hydrodynamic diameter of the mNPs was as described by Chemicell (~ 200 nm), which was confirmed *via* dynamic light scattering, with particles exhibiting an increase in particle negativity as measured by zeta potential demonstrating the successful attachment of penetratin [24]. The NPs appear smaller with TEM, as only the electron-dense core is visible. 

### 3.2. NP Internalisation

#### 3.2.1. ICP-MS Analysis

**Figure 2:**
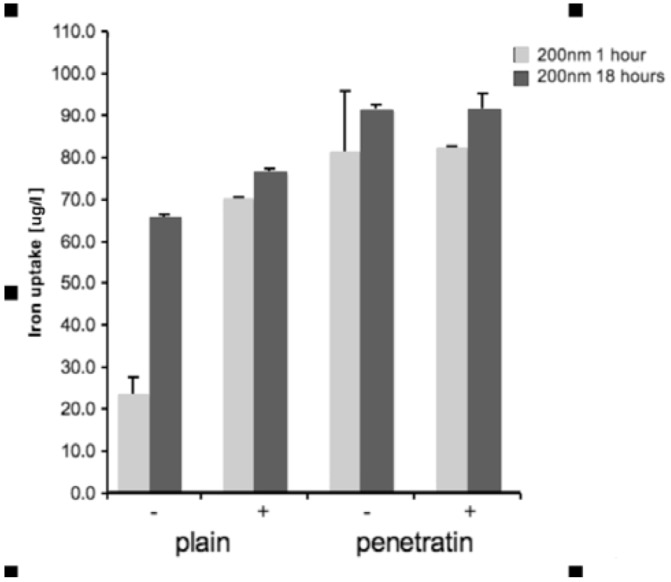
Amount of NP uptake into monolayer cells. Both a magnetic field and the attachment of penetratin significantly increased cellular uptake of the 200 nm NPs (average of n = 5 +/-sd; p < 0.001 for all significant differences described in Section 3.2.1).

The amount of mNPs uptaken by cells was quantified by ICP-MS analysis of iron after both mNPs had been incubated with the cells for both 1 and 18 hours (Figure 2). 

Two key significantly different observations were noted: (i) a MF increased the uptake of plain NPs at 1 hour compared to no MF, whereas the penetratin NPs showed no enhanced uptake with a MF (ii) penetratin increased the uptake of both NPs at 1 hour and 18 hours as compared to plain NPs. In addition it was noted that the penetratin uptake (no MF) was comparable at both time points. 

#### 3.2.2. Fluorescence Microscopy

**Figure 3:**
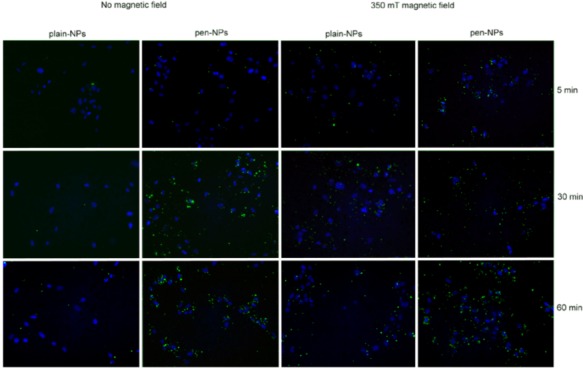
Fluorescence microscopy to identify uptake patterns over time. Uptake of both plain and penetratin NPs +/- magnetic field (MF) over a 60 minute incubation period for the 200 nm NPs (green: NPs, blue: nucleus).

Observations of particle uptake over a 60-minute time period were performed, as ICP-MS results indicated that, aside from the plain without a field, uptake did not increase significantly beyond that point (Figure 3). During the 60-minute period, several key points were noted: (i) uptake was typically increased in a time dependent manor, (ii) penetratin indicated higher uptake levels as compared to plain NPs, (iii) a magnetic field increases uptake and (v) the penetratin NPs appear to be more cell-associated than the plain NPs (clustering around the cell nuclei).

#### 3.2.3. Fluorescent Platereader Analysis

**Figure 4:**
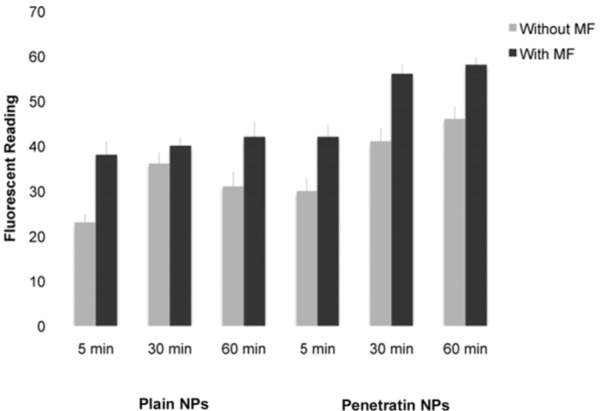
Assessment of 200 nm NP internalisation after 1 hour incubation as measured *via* fluorescent plate reader (average of n = 6 +/-sd; p < 0.001 for all significant differences described in Section 3.2.3).

The cellular uptake of the plain and penetratin functionalised 200 nm NPs was quantified using a fluorescent plate-reader (Figure 4). All results described were significantly different and data confirmed observations in Section 3.2.2: (i) the NP uptake increased in a time dependent manor, (ii) a MF increased the uptake of both NPs compared with without an MF, and (iii) penetratin NPs were more efficiently internalised than plain NPs both +/- MF.

### 3.3. Route of Internalisation

#### 3.3.1. Clathrin and Caveolin qRT-PCT

**Figure 5:**
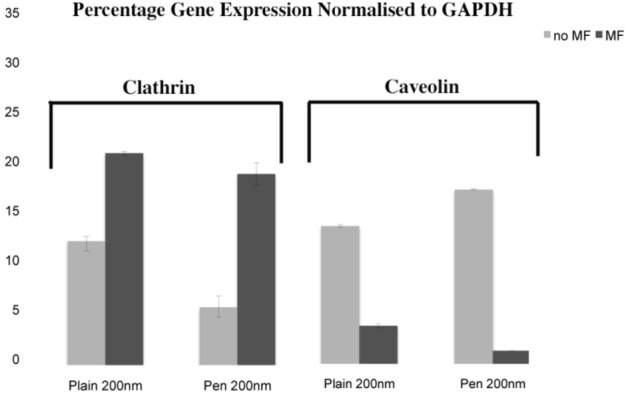
Clathrin and caveolin expression as assessed by qRT-PCR, normalised to GAPDH (n = 3, from two experiments, +/- sd).

The levels of clathrin and caveolin mRNA expression were quantified by qRT-PCR after a 1 hour incubation with the NPs (Figure 5). In the absence of a magnetic field, while both clathrin and caveolin expressions were increased for the plain and penetratin, the caveolin was higher. However this trend was reversed in the presence of a magnetic field, where instead clathrin dominated, and a large decrease in caveolin was noted.

#### 3.3.2. Clathrin and Caveolin Western Blots

**Figure 6:**
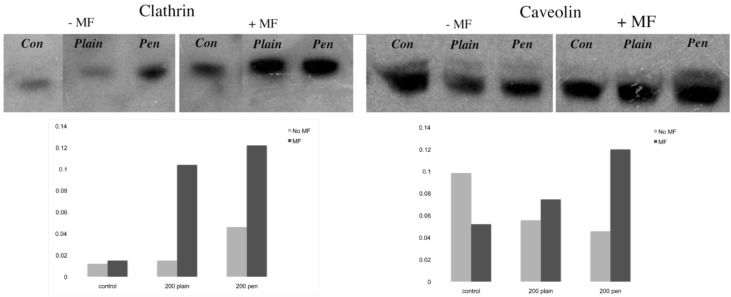
Clathrin and caveolin protein levels as assessed by western blotting, with band intensity analysed *via* Image J.

The amount of clathrin and caveolin protein were assessed by western blot after a 1 hour incubation with each of the NPs (Figure 6). With regards to the clathrin results, the trends for protein expression levels were similar to the PCR results. In the absence of a field, only the penetratin-functionalised NPs demonstrated a slight increase as compared to controls, however when a magnetic field was present, clathrin was strongly increased in both cases. Conversely, the caveolin protein expression did not mirror the PCR data. In this case lower caveolin levels were noted without a field, which was reversed in the presence of a field (the opposite of the PCR data).

### 3.4. Blocking Experiments

#### 3.4.1. Fluorescence Microscopy

**Figure 7:**
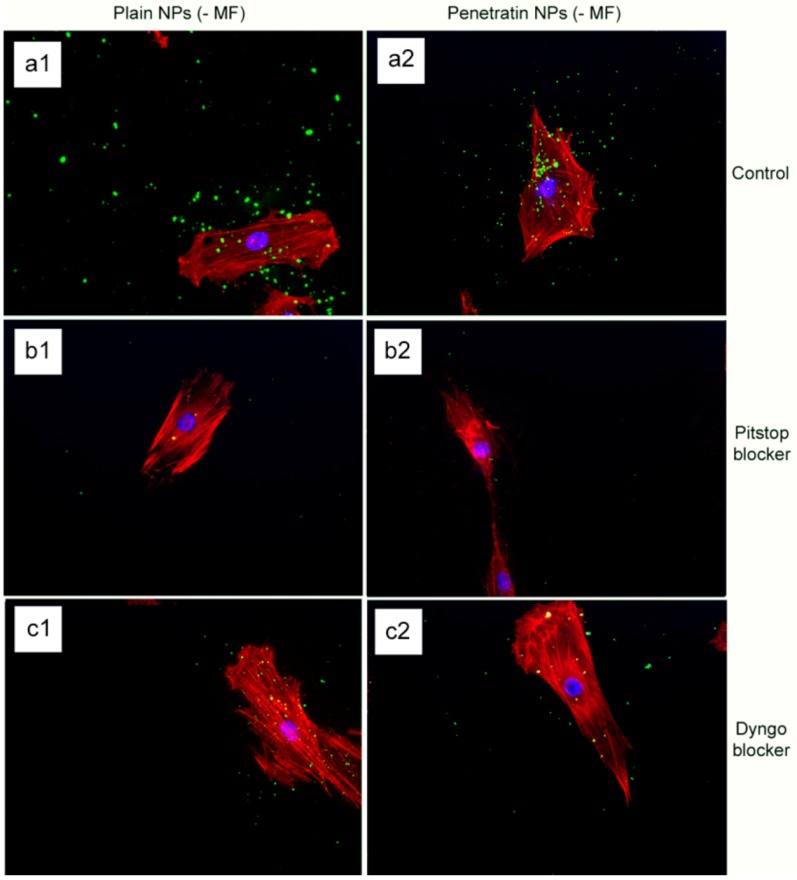
Fluoresence microscopy of plain and penetratin 200 nm particle uptake into cells in the absence of a magnetic field, incubated with either Pitstop or Dyngo endocytosis blockers.

Cell cytoskeleton staining in control cells (without blockers) indicated a similar level of NP uptake both +/- a magnetic field for both the plain and penetratin NPs, where the pentratin NPs appeared more cell associated as compared to the random nature of the plain NPs (Figure 7, a1 and 2). Considering first, the absence of a magnetic field, the presence of the clathrin blocker, Pitstop, resulted in a clear reduction in NP uptake, with NP uptake blocked (Figure 7 b1 and b2). Dyngo also reduced NP uptake, although only partially, as some NPs were still observed (Figure 7 c1 and c2).

**Figure 8:**
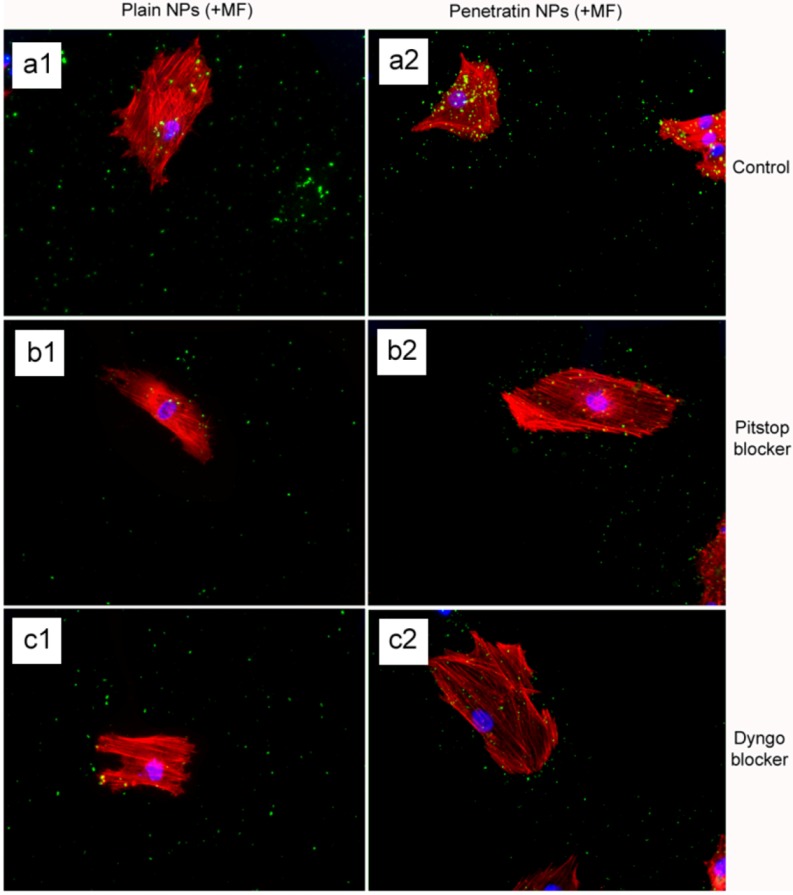
Fluoresence microscopy of plain and penetratin 200 nm particle uptake into cells in the presence of a magnetic field, incubated with either Pitstop or Dyngo endocytosis blockers.

Interestingly, with regards to the presence of a magnetic field, blocking was evident with both the Pitstop and Dyngo, but only partially, as some NPs were still clearly observed (Figure 8 b1 and b2, c1 and c2). This suggests that the magnetic field demonstrated an ability to overcome the potency of both blockers, Pitstop and Dyngo, signifying that either the attractive force of the magnetic field somehow interferes with the action of the blockers, or that NP uptake under a field is using a difference method of uptake.

#### 3.4.2. Transmission Electron Microscopy (TEM)

**Figure 9:**
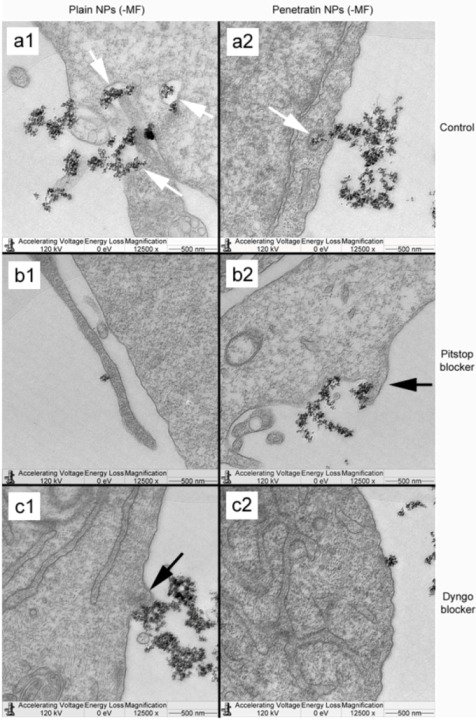
TEM micrographs showing cellular uptake of plain and penetratin functionalised NPs in the absence of a magnetic field. Control cells (no blockers; a1 and a2), cells incubated in Pitstop (b1 and b2) and cells incubated in Dyngo (c1 and c2). Note the cell membrane invaginations and vacuole formations indicative of endocytosis (white arrows) and the membrane extrusions indicative of pinocytosis (black arrows).

TEM was used to locate NPs uptaken into the cell body. In the absence of a field, control cells (no blockers) illustrated uptake of both plain and penetratin NPs through membrane invaginations resulting in NPs localised in vacuoles in the cytoplasm (Figure 9 a1 and a2; see annotated white arrows). When cells were incubated with either Pitstop of Dyngo, both the plain and penetratin NPs appeared to accumulate at the cell membrane, with less evidence of cellular uptake and packaging into vacuoles, and exhibited more membrane protrusions suggesting pinocytosis (Figure 9 b1 and b2, c1 and c2; see annotated black arrows). 

**Figure 10:**
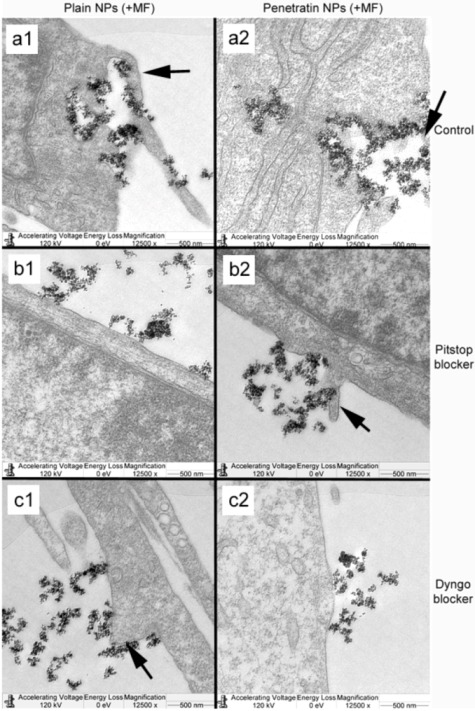
TEM micrographs showing cellular uptake of plain and penetratin functionalised NPs in the presence of a magnetic field. Control cells (no blockers; a1 and a2), cells incubated in Pitstop (b1 and b2) and cells incubated in Dyngo (c1 and c2). Note the appearance of membrane extrusions, particularly in the control cells, indicative of pinocytosis (black arrows).

In the presence of a magnetic field, control cells demonstrated a higher level of NP uptake, with NP loaded vesicles clearly evident packaged and being processed throughout the cell cytoplasm (Figure 9 a1 and a2). With regard to Pitstop and Dyngo, both the plain and penetratin NPs appeared to accumulate at the cell membrane, however in this case, there was evidence of cell membrane extensions encompassing NPs (see annotated arrows in Figure 10), indicative of pinocytosis (Figure 10 b1 and b2, c1 and c2).

#### 3.4.3. ICP-MS

**Figure 11:**
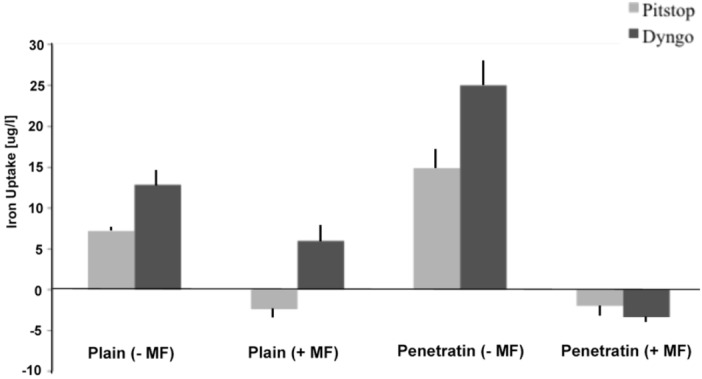
Assessment of NP uptake into cells by ICP-MS levels measured in the cell medium and normalised to control cell cultures (n = 5 from 2 experiments, ± sd).

The amount of NP uptake into the cells, under the influence of two blockers Pitstop and Dyngo, was quantified by ICP-MS analysis (Figure 11). Please note that the measurements are taken from the cell medium and normalised to control cells, thus an increase indicates a higher level of NPs in the medium, thus a reduction in the level entering the cells. The data reflected the blocking results observed in both the previous fluorescence and TEM studies, whereby a reduction in uptake was observed in the absence of a magnetic field, but this blocking was partially reversed in the presence of a field (as indicated by the negative values).

## 4. Conclusions

In this study, we have demonstrated a comprehensive and comparative analysis of cellular uptake of plain and penetratin magnetic NPs in the presence and absence of external magnetic field. The initial part of the paper was designed to measure and compare total uptake into cells. Results clearly showed that the penetratin increased cellular uptake, as expected, and that the uptake was more cell specific compared to plain nanoparticles. Penetratin is a secondary amphipathic CPP that has been studied extensively over the last decade, and its functionalisation with NPs strongly augments the NP uptake. The cell association observed was most likely due to the net penetratin positive charge (due to the presence of abundant appropriate amino acid residues, e.g. arginine) that promotes a strong affinity towards the negative cell plasma membrane. This attraction would contribute to the enhancement in the uptake of penetratin NPs, and may also enable the ability of escaping extensive degradation by endosomes [26]. In addition to penetratin enhancing uptake, a magnetic field also increased NP uptake into cells, particularly within the first hour of culture, with little difference was noted after 18 hours in culture, suggesting an uptake threshold or saturation. Previous general observations showed that magnetic NP uptake increased in the presence of static magnetic fields, as the field physically accelerates the sedimentation of mNPs on the surface of the cell [23,27]. Indeed, this is the mechanism behind the commercially available ‘magnetofection’ techniques (i.e. transfecting using a magnetic field) [28]. 

The second part of the paper was aimed at identifying the possible means of NP uptake, focusing on the two more established routes of uptake: clathrin- and caveolin-mediated endocytosis. Early work had suggested a receptor-independent pathway to be responsible for the penetratin uptake [29]. However subsequent studies have indicated that endocytosis in conjunction with direct translocation to be the actual mechanism of uptake. The presence of arginine and lysine (highly positively charged amino acids) play an essential part in the cell uptake of penetratin [30] alongside the presence of proteoglycans on the cellular surface, which are crucial for its initial interaction with the cells [31]. In this study, plain and penetratin NPs both demonstrated an increase in clathrin (*via* PCR and western blot), highlighting clathrin’s role is NP uptake. Unfortunately the caveolin results were contradictory, however with a cargo size of 200 nm, caveolin uptake is expected to be limited.

To date, there has been little work to elucidate the cellular uptake route under the influence of a magnetic field, despite the frequent use in culture. Previous studies in our group using 500 nm magnetic NPs have suggested that an external static magnetic field may increase the cellular levels of clathrin, thus instigating clathrin-mediated endocytosis of the 500 nm magnetic NPs [23], whilst low caveolin levels were detected. This was again shown here, with 200 nm magnetic NPs, whereby clathrin gene and protein expression were noted higher in the presence of a field. Brief exposure to a static magnetic field (15 minutes, 100 mT) has previously been shown to increase activator protein-1 (AP-1) expression, a transcription factor that regulates gene expression in response to a variety of stimuli, and thus controls various cell processes including proliferation and apoptosis [32]. It may be that a magnetic field also influences endocytosis through such transcription factors.

The final part of the paper was aimed at further study of the use of clathrin in the uptake of both plain and penetratin NPs, with and without a magnetic field by employing specific clathrin endocytosis blockers. Recently published data using Pitstop 2 has clearly shown its blocking impact on clathrin- mediated endocytosis, that selectively impede the association of endocytic ligands with the clathrin terminal domain which is mainly involved in the internalization of various pathogens and growth factors [10]. In this study, all data (fluorescent uptake, TEM and ICP-MS) indicated that Pitstop blocked uptake of both the plain and penetratin mNPs, confirming uptake is *via* clathrin-mediated endocytosis.

The second blocker employed, Dyngo, is known to block dynamin, that causes the scission of the vesicles formed during the internalisation of NPs. Dyngo was earlier extensively studied, as an inhibitor of *Botulinum* neurotoxins, that mainly causes paralytic symptoms of the botulism, and this blocker has been proved to hinder the internalisation of these neurotoxins in the nerve terminals, that mainly enter neurons through synaptic vesicles and via clathrin-coated pits [25]. However, in this study, this interfering property of dyngo during endocytosis was evident to a less extent, as partial blocking in the uptake was observed.

The blocking of NP uptake observed with Pitstop and Dingo was reversed under the presence of a magnetic field, as both the penetratin as well as plain NPs were noted in the cell (indicated by fluorescence, TEM and ICP-MS). This may be due to several possibilities. Data from Section 3.3 demonstrated an increase in clathrin in the presence of a magnetic field, thus perhaps the field is somehow stimulating cell clathrin synthesis to an extent whereby levels overwhelm the blockers. Alternatively, the strong magnetic force that pulls the NPs inside the cell may be great enough to totally overcome the clathrin blocking activity, by directly pulling mNPs through the cell plasma membrane, a theory supported by previous publications [33,34]. A third possibility may be that the field is instigating a different uptake pathway, such as an increase in uptake *via* macropinocytosis. Certainly, when considering the TEM images, there was clear evidence for pinocytosis in control cells under a magnetic field (Figure 9 a1 and a2), which was not as obvious without a field. A recently published study focusing on the magnetically enhanced uptake of 50 nm magnetic silica mesoporous NPs identified clathrin as the main route of uptake (*via* blocking experiments; 30 mins incubation, 0.1T), however the blocking was also reversed under the influence of a magnetic field [35]. The authors surmised that the enhanced uptake observed under a field depended on the field properties, that the field was strong enough to essentially pull the NPs through the membrane, based on reported calculation of magnetic NPs begin pulled through model membranes [34].
